# White Matter Microstructural Correlates of Auditory Brainstem Responses in Patients With Charcot‐Marie‐Tooth Disease

**DOI:** 10.1002/brb3.71142

**Published:** 2026-01-25

**Authors:** Peng Wang, Zhiyuan An, Yan Huang, Wen Qi, Xu Han, Yuqi Xia, Zhe Chen, Zhenghan Yang, Pengfei Zhao, Yuhe Liu

**Affiliations:** ^1^ Department of Radiology, Beijing Luhe Hospital Capital Medical University Beijing China; ^2^ Department of Radiology, Beijing Friendship Hospital Capital Medical University Beijing China; ^3^ School of Mathematics and Statistics Shanxi University Taiyuan China; ^4^ Department of Otolaryngology, Head and Neck Surgery, Beijing Friendship Hospital Capital Medical University Beijing China

**Keywords:** auditory brainstem response, Charcot‐Marie‐Tooth disease, diffusion tensor imaging, micro‐structural abnormalities, white matter

## Abstract

**Introduction:**

Some patients with Charcot‐Marie‐Tooth disease (CMT) exhibit prolonged auditory brainstem response (ABR) latencies or abnormal waveforms, suggesting potential damage to the peripheral auditory nerve or central auditory pathways. Diffusion tensor imaging (DTI), a non‐invasive neuroimaging technique, can detect the integrity and functional properties of white matter structures with high sensitivity. However, research on the association between DTI characteristics and ABR changes in patients with CMT remains relatively limited, and whether both modalities reflect synergistic damage to central‐peripheral nerve axons or myelin sheaths remains unclear. In this study, we aimed to analyze cerebral white matter microstructural abnormalities in patients with CMT using DTI and explore their correlation with ABR, thereby exploring the pathophysiological mechanisms of the central auditory pathway in patients with CMT.

**Methods:**

This study included 14 patients with CMT and 14 healthy controls. DTI data were acquired using a 3.0T MRI scanner. Fractional anisotropy (FA), mean diffusivity (MD), axial diffusivity (AD), and radial diffusivity (RD) were calculated. The latencies and interpeak latencies of the auditory pathway were measured using ABR. DTI metrics were compared between the two groups, and the relationship between DTI parameters and ABR results was analyzed in patients with CMT.

**Results:**

Compared with the healthy controls, patients with CMT exhibited significantly decreased FA values and significantly increased MD, AD, and RD values in brain regions *p* < 0.05), including the occipital part of the corona radiata, inferior longitudinal fasciculus, anterior thalamic radiation, and inferior fronto‐occipital fasciculus. ABR interpeak latencies correlated positively with FA in the left inferior longitudinal fasciculus and negatively with AD. Three participants did not complete the ABR test. ABR latencies in CMT patients were significantly correlated with AD values in the anterior thalamic radiation and corpus callosum (*p* < 0.05).

**Conclusion:**

Abnormal central white matter microstructure (axonal degeneration, demyelination) in patients with CMT may lead to auditory pathway dysfunction by impairing neural conduction efficiency. The multimodal correlation analysis of DTI and ABR provides new insights into the mechanism of central nervous system involvement in CMT, suggesting its potential as a clinical biomarker.

## Graphical abstract

We quantitatively analyzed the cerebral white matter microstructure in patients with CMT using DTI. We identified cerebral white matter microstructural abnormalities in CMT patients, including the FMA and ILF. These alterations correlated with ABR interpeak latencies, suggesting impaired central auditory processing due to disrupted axonal‐myelin integrity.

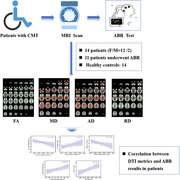

## 1 Introduction

Charcot‐Marie‐Tooth disease (CMT) is an inherited motor and sensory neuropathy caused by various gene mutations, with an incidence rate of approximately 1 in 2500 persons. Its primary symptoms include walking difficulties, sensory loss, and foot deformities, amongst others (Okamoto and Takashima 2023). The proteins encoded by the mutated genes are expressed in the myelin sheath or axons of peripheral nerves, leading to defects in peripheral nerve myelination or axonal dysfunction, such as reduced conduction velocity and decreased response amplitudes (Hong et al. 2017; Cheah et al., 2021).

Demyelination in patients with CMT is not confined to the peripheral nerves; the white matter microstructure in the central nervous system (CNS) may also be affected. Studies using diffusion tensor imaging (DTI) have reported microstructural alterations in the white matter in the CNS of patients with CMT; these include abnormalities in the cerebellum, corpus callosum, and corona radiata (Pontillo et al. 2020; Hwang et al. 2021). DTI, as a noninvasive, in vivo technique for white matter tractography, enables sensitive assessment of the structural integrity and tissue properties of white matter through the quantification of multiple diffusion parameters. The primary metrics include fractional anisotropy (FA), mean diffusivity (MD), axial diffusivity (AD), and radial diffusivity (RD). Specifically, FA reflects the directional coherence of white matter fibers and integrity; MD characterizes the overall magnitude of water diffusion, indicating tissue density and structural barriers; AD primarily captures the structural integrity of axons; and RD is specifically associated with changes in myelin integrity (Tae et al. 2018; Behler et al. 2023). The combined use of these parameters provides multidimensional insights into fiber integrity, myelination status, and axonal injury, thereby offering comprehensive imaging evidence for elucidating potential trans‐synaptic degeneration and central‐peripheral nervous system interactions in CMT.

Recent evidence from a mouse model of CMT indicated that transient auditory nerve demyelination can lead to hidden hearing loss, accompanied by prolonged interpeak latencies in the auditory brainstem response (ABR) (Cassinotti et al. 2024). This phenomenon suggested that myelin pathology may directly impair signal transmission efficiency along the auditory brainstem pathway. Further investigation revealed that changes in myelin thickness are significantly correlated with shortened ABR latencies, demonstrating that structural remodeling of myelin can directly optimize nerve conduction velocity and ABR (Stancu et al. 2024). Concurrently, ABR serves as a key electrophysiological method for assessing auditory pathway function, reflecting signal conduction efficiency from the cochlear nerve to the brainstem level (Habib and Habib 2021). Notably, some patients with CMT exhibit prolonged ABR latencies or abnormal waveforms, suggesting potential damage to the peripheral auditory nerve or central auditory pathways (Neijenhuis et al. 2003; Mills et al. 2024).

Techniques to assess the white matter microstructure offer new perspectives for investigating abnormalities in the auditory brainstem pathway. Analysis of patients with neurofibromatosis type 1 revealed a significant reduction in the apparent fiber density in their ascending auditory brainstem tracts (Rance et al. 2021). In addition, specific correlations exist between properties of the white matter microstructure in the brainstem (such as MD and FA) and sensory characteristics (including auditory), providing a structural foundation for understanding the neural basis of sensory deficits (Surgent et al. 2022). However, research on the association between DTI characteristics and ABR changes in patients with CMT remains relatively limited, and it is still unclear whether both modalities reflect combined impairment of the central‐peripheral nerve axons or myelin sheaths.

Therefore, we aimed to systematically analyze microstructural differences in cranial white matter between patients with CMT and healthy controls (HCs) using DTI technology. Furthermore, we explored potential correlations between DTI parameters and ABR indicators to explore the patterns of central auditory pathway damage in patients with CMT. By integrating multimodal neuroimaging and neurophysiological data through the investigation of DTI parameters and ABR in patients with CMT, this research is expected to provide new insights into the mechanisms of CNS involvement in CMT and offer more comprehensive biomarkers for clinical assessment.

## 2 Materials and Methods

### 2.1 Patients

We prospectively enrolled patients with CMT diagnosed at the Beijing Friendship Hospital, Capital Medical University, from January 2024 to December 2024. Patients with CMT were included if they (1) were diagnosed with CMT based on comprehensive clinical assessment and genetic testing, (2) underwent cranial DTI examination, (3) underwent ABR examination, and (4) were aged ≥18 years old. The general exclusion criteria for patients with CMT and healthy controls (HCs) were (1) pregnancy or lactation; (2) comorbid neurological, cardiovascular, cerebrovascular, or endocrine system diseases; (3) history of substance abuse or alcohol dependence; (4) poor‐quality MRI data (significant susceptibility artifacts or incomplete raw data); and (5) significant brain lesions or white matter hyperintensities. CMT was classified into different subtypes according to the genetic characteristics, clinical manifestations, and electrophysiological and pathological features of the patients (Braathen GJ 2012). Age‐ and sex‐matched HCs were recruited during the same period. The Hospital Ethics Committee approved this study (Approval No. 2024‐P2‐449‐01), and all participants provided written informed consent before the examination.

### 2.2 MRI Data Acquisition

Scanning was performed using a 3.0T MRI scanner (Siemens, Prisma, Germany), and a single MRI technologist performed all examinations. The scan included the whole brain range from the top of the skull to the base. DTI was acquired using the following parameters: Echo‐planar imaging sequence, repetition time (TR) = 8500 ms, echo time (TE) = 63 ms, flip angle = 90°, field of view (FOV) = 240 × 240 mm^2^, matrix size = 224 × 224, slice thickness = 2 mm, slice gap = 0 mm, b‐value = 1000 s/mm^2^, diffusion‐encoding directions = 64, structural imaging (three‐dimensional [3D] T1‐weighted). The following 3D T1WI parameters were used: TR = 2530 ms, TE = 2.98 ms, matrix size = 512 × 512, FOV = 256 × 256 mm2, flip angle = 7°, slice thickness = 1 mm.

### 2.3 Data Processing and Analysis

Format Conversion: Imaging data were converted to NIFTI format using *dcm2niix*, and image quality was visually assessed using *MRIcroGL*.

Preprocessing: DTI data underwent noise reduction and artifact correction using MRtrix tools (https://www.mrtrix.org/). The pre‐processing steps include noise reduction, Gibbs ringing artifact removal, and motion and eddy current correction using FSL's eddy tool (https://fsl.fmrib.ox.ac.uk/fsl/fslwiki/) using the dwifslpreproc command in MRtrix, with subsequent B‐vector rotation based on transformation matrices. N4 bias field correction was performed using advanced normalization tools to address intensity inhomogeneity.

Microstructural metric calculation: Brain masks were extracted from the b0 images using FSL's BET tool, and diffusion tensors were estimated using the dwi2tensor command in MRtrix. FA, MD, RD, and AD maps were derived.

Cross‐Sectional Statistical Analysis: Tensor‐based spatial statistics (TBSS) in FSL were employed. Individual FA maps were non‐linearly registered to the FMRIB58_FA template, the mean FA skeleton (threshold: FA > 0.2) was generated, and individual FA maps were projected onto this skeleton. Non‐FA metrics (MD, AD, RD) were projected onto the same skeleton using tbss_nonFA.

Statistical analysis of microstructural metrics was performed using the randomize tool in FSL, employing a two‐sample t‐test design with age and sex as covariates. The analysis included 5,000 permutations with statistical significance determined by threshold‐free cluster enhancement correction at *p* ≤ 0.05. Results were reported according to the Johns Hopkins University White‐Matter Tractography Atlas.

### 2.4 ABR Test

ABR was recorded using click stimuli filtered at 100–3000 Hz, delivered at a rate of 21.1/s with 1024 sweeps averaged over a 10‐ms analysis window. Skin degreasing was performed at all contact sites before electrode placement to maintain inter‐electrode impedance below 4 kΩ. The active electrodes were positioned at the mid‐frontal hairline, reference electrodes on the bilateral mastoids, and the ground electrode on the glabella. Stimuli were presented at 80 dB nHL, and waveforms were analyzed for clearly identifiable peaks to record absolute latencies of waves I, III, and V, along with interpeak latencies (I–III, III–V, and I–V).

### 2.5 Statistical Analysis

Statistical analysis was performed using the SPSS 25.0 software. Normally distributed continuous data are expressed as mean ± standard deviation (mean ± SD). Differences in DTI metrics between the CMT and HC groups were compared using two‐sample t‐tests with false discovery rate (FDR) correction at the voxel level. Pearson correlation analysis was employed to examine relationships between FA, MD, and AD, and between RD values and ABR results within the CMT group. Statistical significance was set at *p* < 0.05.

## 3 Results

### 3.1 Participants’ Characteristics

This study enrolled 14 patients with CMT (two males and 12 females; mean age 37.93 ± 13.47 years) and 14 matched controls. No significant differences were observed between the groups regarding age or sex (*p* > 0.05). The CMT subtypes included CMT1 (n = 8), CMT2 (n = 3), CMTX (n = 2), and CMT4 (n = 1). Of the patients with CMT, three did not undergo ABR testing. In these individuals, the latencies of waves I, II, and V in the ABR test were 1.49 ± 0.17 ms, 3.68 ± 0.23 ms, and 5.59 ± 0.54 ms, respectively. These details are shown in Table 1.

**TABLE 1:** Participants’ demographic and clinical data.

	CMT (n = 14)	Controls (n = 14)	*p* value
Ages (years)	37.93 ± 13.47	38.29 ± 12.69	0.94
Sex			
Female/Male	12/2	12/2	1.00
Types (n, %)			
CMT1	8 (57.14%)		
CMT2	3 (21.43%)		
CTMX	2 (14.29%)		
CMT4	1 (7.14%)		
ABR (ms)			
Wave I latency	1.49 ± 0.17		
Wave III latency	3.68 ± 0.23		
Wave V latency	5.59 ± 0.54		
I‐III interpeak latency	2.19 ± 0.20		
III‐V interpeak latency	1.91 ± 0.38		
I–V interpeak latency	4.10 ± 0.52		

**Abreviations**:ABR, auditory brainstem response; CMT, Charcot‐Marie‐Tooth disease

### 3.2 TBSS Analysis of FA

**FIGURE 1:**
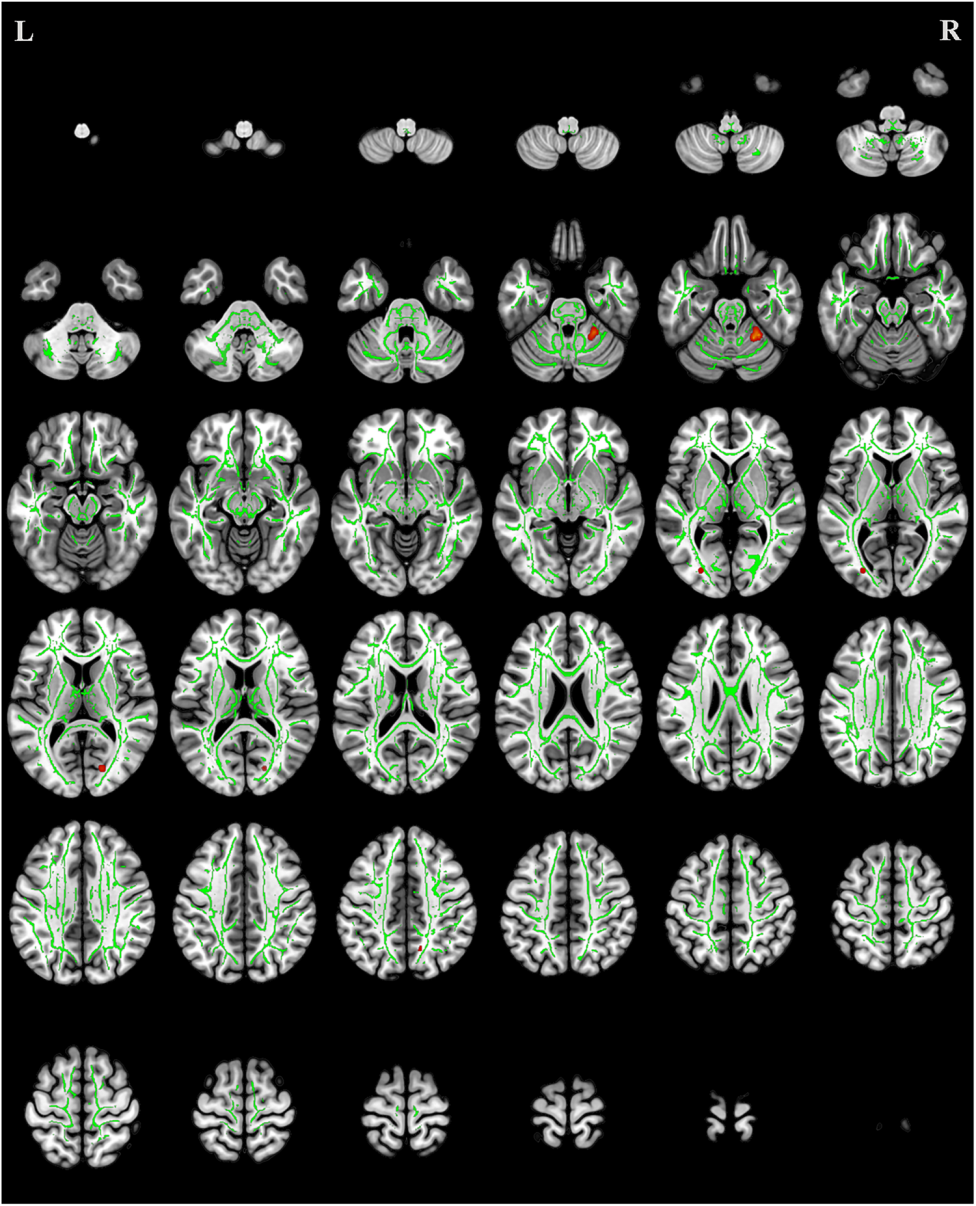
Regions of reduced fractional anisotropy in patients with Charcot‐Marie‐Tooth disease (red) compared to healthy controls (*p* < 0.05, uncorrected). The green areas represent the mean fractional anisotropy skeleton used for tract‐based spatial statistics.

Compared with the HCs, the CMT group exhibited significantly reduced FA values (*p* < 0.05, uncorrected) in multiple white matter tracts, including forceps major (FMA), right cingulum (cingulate gyrus), and left inferior longitudinal fasciculus (ILF) (Table 2, Figure 1).

**TABLE 2:** White matter with decreased fractional anisotropy.

Cluster size (voxels)	MNI center of mass			Side	Brain region	*p* value
	X	Y	Z			
4	18	−77	11	/	Forceps major	0.005
2	−28	−81	4	L	Inferior longitudinal fasciculus	<0.001
1	12	−59	45	R	Cingulum (cingulate gyrus)	0.009

**Abbreviations**: L/R, left/right; MNI, Montreal Neurological Institute

### 3.3 TBSS Analysis of MD

**FIGURE 2:**
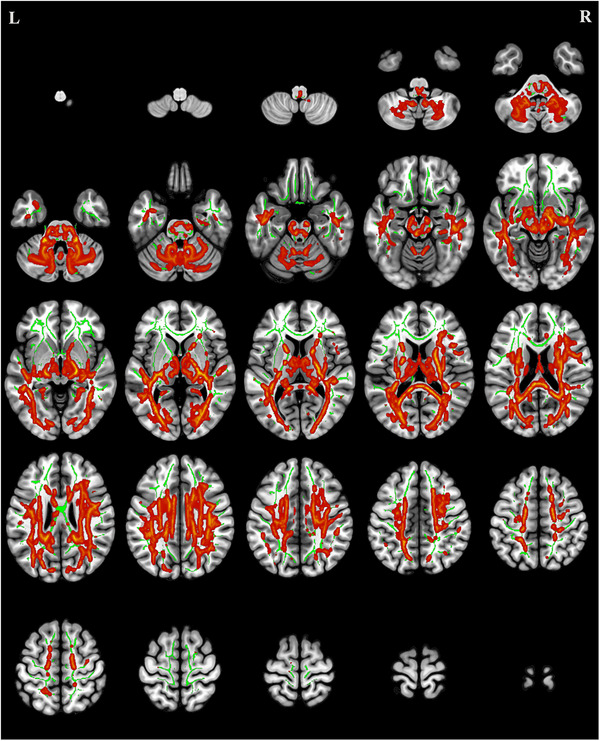
Regions of increased mean diffusivity in patients with Charcot‐Marie‐Tooth disease (red) compared to healthy controls (*p* < 0.05, false discovery rate [FDR] correction). The green areas depict the mean fractional anisotropy skeleton from tract‐based spatial statistics.

Compared with the HCs, the CMT group exhibited significantly increased MD values (*p* < 0.05 with cluster‐level FDR correction) in multiple white matter tracts, including the left anterior thalamic radiation (ATR), bilateral ILF, and left cingulum (hippocampus) (Table 3, Figure 2).

**TABLE 3:** White matter with increased mean diffusivity.

Cluster size (voxels)	MNI center of mass			Side	Brain region	*p* value
	X	Y	Z			
141	−20	9	14	L	Anterior thalamic radiation	0.022
116	59	−9	0	R	Inferior longitudinal fasciculus	0.020
111	−41	−73	13	L	Inferior longitudinal fasciculus	0.007
104	−23	−45	0	L	Cingulum (hippocampus)	0.017

**Abbreviations**: L/R, left/right; MNI, Montreal Neurological Institute

### 3.4 TBSS Analysis of AD

**FIGURE 3:**
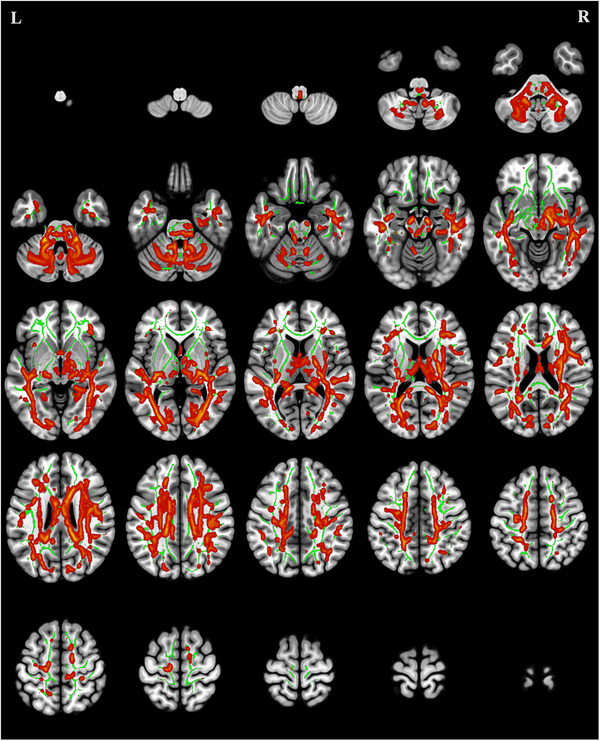
Regions of elevated axial diffusivity in patients with Charcot‐Marie‐Tooth disease (red) compared to healthy controls (*p* < 0.05, FDR correction). The green areas depict the mean fractional anisotropy skeleton derived from tract‐based spatial statistics.

Compared with the HCs, the CMT group demonstrated significantly elevated AD values (p < 0.05 with cluster‐level FDR correction) in multiple white matter tracts, including the right corticospinal tract, left ATR, FMA, left uncinate fasciculus, bilateral superior longitudinal fasciculi, right inferior fronto‐occipital fasciculus (IFOF), and left ILF (Table 4, Figure 3).

**TABLE 4:** White matter with increased axial diffusivity.

	Cluster size (voxels)	MNI center of mass			Side	Brain region	*p* value
		X	Y	Z			
	19887	28	−45	−2	R	Corticospinal tract	0.001
	11421	−31	−62	32	L	Anterior thalamic radiation	0.002
	602	15	−97	8	/	Forceps major	0.010
331	559	−42	18	17	L	Uncinate fasciculus	0.015
	296	−31	32	27	L	Anterior thalamic radiation	0.012
	171	−53	−13	33	L	Superior longitudinal fasciculus	0.004
	135	−54	−29	6	L	Superior longitudinal fasciculus	0.028
	126	33	−21	39	R	Superior longitudinal fasciculus	0.025
	126	−23	−33	8	L	Anterior thalamic radiation	0.006
	124	39	38	−8	R	Inferior fronto‐occipital fasciculus	0.011
	120	−44	0	−31	L	Inferior longitudinal fasciculus	0.015

**Abbreviations**: L/R, left/right; MNI, Montreal Neurological Institute

### 3.5 TBSS Analysis of RD

**FIGURE 4:**
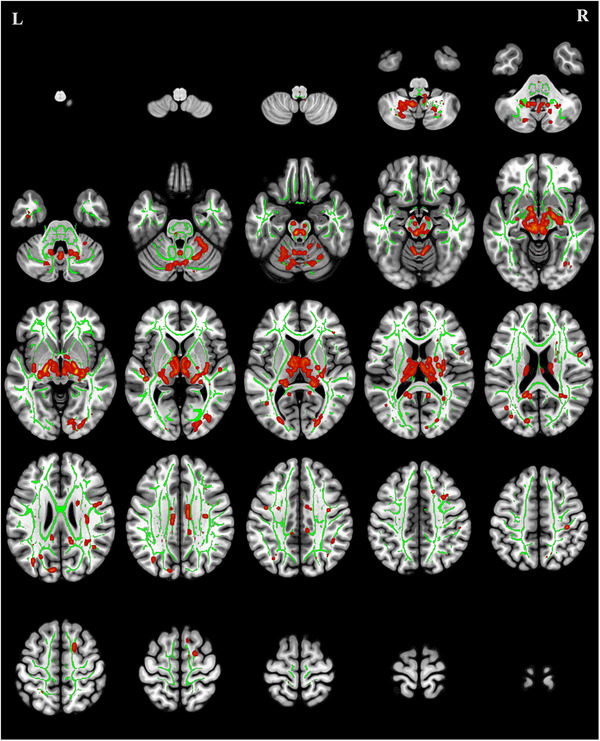
Regions of increased radial diffusivity in patients with Charcot‐Marie‐Tooth disease (red) versus healthy controls (*p* < 0.05, FDR correction). The green areas represent the mean fractional anisotropy skeleton from tract‐based spatial statistics analysis.

Compared with the HCs, the CMT group exhibited significantly increased RD values (p < 0.05 with cluster‐level FDR correction) in multiple white matter tracts, including the left ATR, right ILF, right IFOF, and FMA (Table 5, Figure 4).

**TABLE 5:** White matter with increased radial diffusivity.

Cluster size (voxels)	MNI center of mass			Side	Brain region	*p* value
	X	Y	Z			
3117	−6	−17	−13	L	Anterior thalamic radiation	0.004
198	24	−15	−9	R	Inferior longitudinal fasciculus	0.003
106	35	−9	0	R	Inferior fronto‐occipital fasciculus	0.027
100	−12	−84	33	/	Forceps major	0.015

**Abbreviations**: L/R, left/right; MNI, Montreal Neurological Institute

### 3.6 Correlation Between DTI Metrics and ABR Results in Patients With CMT

**FIGURE 5:**
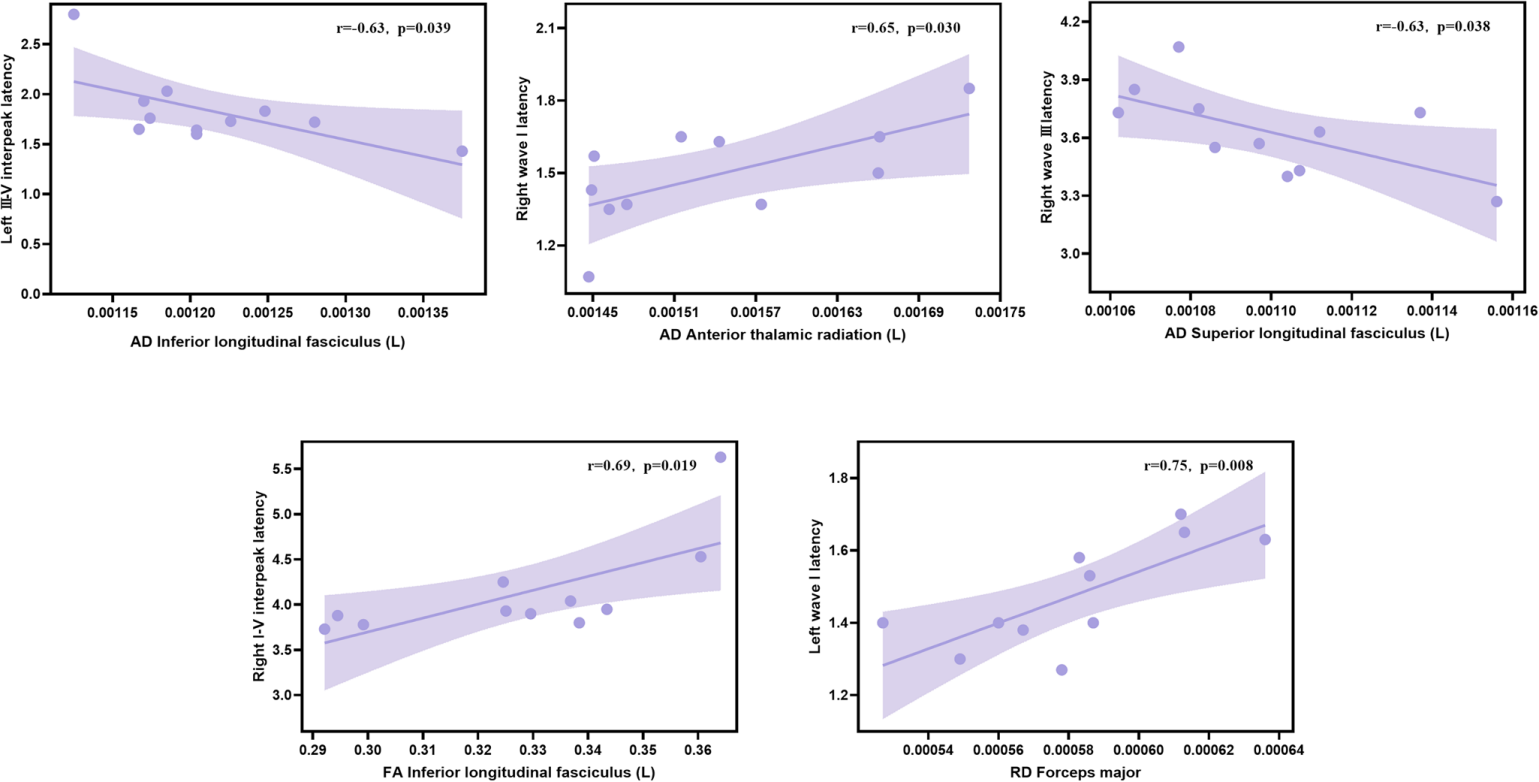
Correlation between diffusion tensor imaging metrics and auditory brainstem response results in patients with Charcot‐Marie‐Tooth disease.

Significant correlations were observed between ABR parameters and DTI metrics in patients with CMT (Figure 5). A positive correlation was observed between right‐ear I‐V interpeak latency and FA in the left ILF (r = 0.69, *p* = 0.019). A negative correlation was observed between left‐ear III‐V interpeak latency and AD in the left ILF (r = −0.63, *p* = 0.039). A positive correlation was observed between right‐ear wave I latency and AD in the left ATR (r = 0.65, *p* = 0.030). A negative correlation was observed between right‐ear wave III latency and AD in the left superior longitudinal fasciculus (r = −0.63, *p* = 0.038). A positive correlation was observed between left‐ear wave I latency and AD in the FMA (r = 0.75, *p* = 0.008).

## 4 Discussion

In this study, we quantitatively analyzed the cerebral white matter microstructure in patients with CMT using DTI. We identified microstructural abnormalities in several regions, including the FMA, ILF, left ATR, and right IFOF, indicating compromised axonal‐myelin integrity and structural disorganization of white matter tracts. Furthermore, correlation analyses revealed significant associations between ABR or interpeak latencies and DTI metrics in specific white matter regions. These findings suggest that microstructural damage within central auditory pathways may impair auditory information processing by reducing synaptic transmission efficiency and abnormal neural synchronization, ultimately disrupting spatiotemporal encoding mechanisms.

We identified reduced FA and elevated AD and RD in the FMA and ILF of patients with CMT. The FMA constitutes a critical commissural pathway integrating visual information between cerebral hemispheres (Huang et al. 2023). Concurrently, the ILF facilitates visual processing and visually guided decision‐making through its connections that span the occipital, temporo‐occipital, and anterior temporal regions (Yuki et al. 2020; Zemmoura et al. 2021). These microstructural alterations may reflect compensatory reorganization of balance function in response to proprioceptive‐kinetic impairment secondary to peroneal muscle atrophy. Given the essential role of vision in postural stability (Schoenmaekers et al. 2025), the observed white matter changes likely represent neuroplastic adaptations to augment visual compensation for compromised somatosensory input. This interpretation aligns with the report of Grosse et al. (2020) on impaired visual‐constructive abilities in patients with CMT, providing behavioral validation of visual pathway dysfunction. Converging evidence supports widespread balance‐system remodeling: Hwang et al. (2021) documented reduced cerebellar white matter volume with decreased FA, increased RD, and diminished AD in patients with CMT. Furthermore, consistent with our observations of pan‐cerebral microstructural disruption, Lee et al. (2017) demonstrated abnormal white matter integrity (reduced FA and elevated RD) across multiple CMT subtypes beyond CMT1A.

The ATR, a white matter tract connecting the dorsal thalamus and the prefrontal cortex that is involved in emotional regulation (Wan et al. 2024; Zou et al. 2024) through the anterior limb of the internal capsule, mediates attention, executive functions, working memory (Ferris et al. 2022), and emotional regulation (Nenadić et al. 2025). We observed elevated MD, AD, and RD values in the left ATR of patients with CMT, suggesting potential disruptions in affective and cognitive processing. This aligns with the report of Grosse et al. (2020) on emotional abnormalities in patients with CMT and documented alterations in functional connectivity: reduced dorsal attention network connectivity and enhanced salience network connectivity in patients with CMT1A (Pontillo et al. 2021). Concurrently, we detected axonal and myelin abnormalities in the right IFOF. This critical language pathway originates in the inferior frontal lobe, extends to the occipital lobe, and interfaces with parietal regions through lateral insular connections, supporting language processing and comprehension (Conner et al. 2018; Eze et al. 2024). The findings of Pontillo et al. (2021) regarding diminished functional connectivity within language networks further substantiate cerebral reorganization in CMT.

The ABR assesses auditory pathway integrity and conduction velocity from the cochlea to the brainstem. Previous studies report prolonged ABR latencies, waveform abnormalities (Giuliani et al. 2019), and auditory processing deficits in some patients with CMT (Rance et al. 2012). Choi et al. found preserved speech perception in quiet environments among patients with CMT1A (Choi et al. 2018); however, they demonstrated significant speech‐in‐noise deficits (Choi et al. 2020), with variations across CMT subtypes. Supporting evidence comes from mouse models of CMT1A showing cochlear synaptopathy features, including altered compound action potentials (Cassinotti et al. 2024). Hearing thresholds remain normal in most CMT subtypes; however, mild hearing loss typically occurs in patients with CMT4C (Sivera et al. 2017; Mills et al. 2024). This hidden hearing loss likely stems from auditory nerve demyelination, which impairs neural conduction. Our study reveals significant correlations between ABR interpeak intervals and left ILF FA/AD values, as well as between ABR latencies and regional AD values. These findings suggest that white matter abnormalities disrupt auditory pathway conduction in CMT, positioning AD as a potential biomarker for monitoring auditory pathway integrity in patients with CMT.

### 4.1 Limitations

This study has some limitations. First, given the rarity of the disease, the cohort was limited, and this may have affected the statistical power and generalizability of the results. Because of the limited sample size, subgroup analyses by CMT subtype could have introduced bias. Therefore, we solely focused on CMT as a whole and did not perform an in‐depth investigation of abnormalities in the white matter microstructure or the pathophysiological mechanisms of the central auditory pathway across subtypes. In future studies, we plan to enroll patients with various CMT subtypes for more detailed exploration. Second, the reported FA results were not corrected for multiple comparisons. Therefore, subsequent research should adopt improved correction methodologies to enhance reliability. Third, the ABR dataset was incomplete because three participants did not complete the test, and this may have influenced the statistical power and generalizability of the findings. Finally, the absence of ABR data for HCs limited comprehensive interpretation of neurophysiological alterations specific to CMT.

## 5 Conclusion

This study demonstrates widespread cerebral white matter microstructural abnormalities in patients with CMT, characterized by reduced FA and elevated MD, AD, and RD values. These alterations predominantly affect key white matter tracts, including the FMA, ILF, ATR, and IFOF. Significant correlations between DTI parameters and ABR latencies/interpeak intervals further indicate that microstructural damage impairs auditory information transfer efficiency, supporting the proposed central‐peripheral nervous system co‐pathology in CMT.

## Author Contributions

**Peng Wang**: conceptualization, data curation, formal analysis, methodology, writing – original draft. **Zhiyuan An**: data curation, formal analysis, investigation. **Yan Huang**: conceptualization, project administration, resources, supervision, writing – review and editing. **Wen Qi**: data collection, validation, visualization. **Xu Han**: data curation, software. **Yuqi Xia**: quality control, validation. **Zhe Chen**: quality control, validation. **Zhenghan Yang**: quality control, validation. **Pengfei Zhao**: conceptualization, supervision, writing – review and editing. **Yuhe Liu**: conceptualization, funding acquisition, supervision, writing – review and editing.

## Funding

This work was supported by the China Disabled Persons' Federation (Grant No. 2024CDPFHS‐02), Xicheng District Science and Technology Special Project Plan (Grant No. XCSTS‐SD2024‐05), the National Natural Science Foundation of China (Grant Nos. 82171886 and 82471158), and the Natural Science Foundation of Beijing Municipality (Grant No. 7222301).

## Ethics Statement

This study was approved by the Ethics Committee of Beijing Friendship Hospital, Capital Medical University (Approval No. 2024‐P2‐449‐01). Our study adhered to the Declaration of Helsinki.

## Data Availability

The data that support the findings of this study are available from the corresponding author upon reasonable request.
